# Soil Heavy Metal Pollution and Risk Assessment in Shenyang Industrial District, Northeast China

**DOI:** 10.1371/journal.pone.0127736

**Published:** 2015-05-21

**Authors:** Xudong Jiao, Yanguo Teng, Yanhong Zhan, Jin Wu, Xueyu Lin

**Affiliations:** 1 College of Water Sciences, Beijing Normal University, 100875, Beijing, People Republic of China; 2 Engineering Research Center of Groundwater Pollution Control and Remediation, Ministry of Education of China, 100875, Beijing, People Republic of China; University of Vigo, SPAIN

## Abstract

To investigate the soil heavy metal pollution characteristics and ecological risk factors, 42 samples and six typical soil profiles were collected from the Shenyang industrial district in northeast China and were analyzed for contents of titanium (Ti), copper (Cu), lead (Pb), zinc (Zn), cobalt (Co), nickel (Ni), chromium (Cr) and arsenic (As). Through statistical analysis, it was found that the mean concentrations were higher than their background values (Ti = 4.77>3.8g/kg, Cu = 33.75>22.6 mg/kg, Pb = 45.95>26 mg/kg, Zn = 81.54>74.2 mg/kg, Co = 12.91>12.7 mg/kg, Ni = 32.26>26.9 mg/kg, Cr = 83.36>61 mg/kg and As = 13.69>11.2 mg/kg) but did not exceed their corresponding pollution limits for the Chinese Environmental Quality Standard for Soils (State Environmental Protection Administration of China, 1995). There were contamination hotspots that may be caused by human activities such as smelting plants and sewage irrigation. The Enrichment Factor and Ecological Risk Index were used to identify the anthropogenic contamination and ecological risks of heavy metals. Soil in the study area could be considered lightly or partially polluted by heavy metals. According to clustering analysis, distinct groups of heavy metals were discriminated between natural or anthropogenic sources.

## Introduction

Soils serve as the most important sink for heavy metal pollutants in terrestrial ecosystems[1] and soil heavy metal pollution is a worldwide problem[2]. It is generally considered that heavy metals originate from two primary sources: natural background sources and anthropogenic inputs including metalliferous mining and industries, agrochemicals and mineral fertilizers, vehicle exhaust, sewage sludge and industrial wastes[3]. With the rapid industrialization and urbanization of China over the last two decades, heavy metal pollution in urban and agricultural soils has become significant. High concentrations of heavy metals in surface soil can threaten human health via inhalation, ingestion and dermal contact absorption[4,5]. Heavy metals in deep soil may result in groundwater contamination[6,7]. Soil heavy metal pollution characteristics and ecological risks are the foundation of soil environmental quality assessment.

Shenyang, the administrative center of Liaoning Province and the largest industrial city in Northeast China is known as an industrial base, with heavy machinery and manufacturing as the major industries. Shenyang has used industrial sewage containing a lot of heavy metals in farmland irrigation resulting in a large area of severely contaminated soil. With rapid economic development, soil environmental quality has severely deteriorated and soil heavy metal pollution is one of the most serious problems in Shenyang. Various industrial activities contribute heavy metals to the soil environment directly or indirectly through the release of solid wastes, waste air, and wastewater[8,9]. Furthermore, any contamination of soils could cause groundwater contamination because of the mobility of the pollutants in soils [10,11]. The identification of heavy metal pollution characteristics and ecological risks in the study area would offer essential information for the monitoring and assessment of soils in Shenyang.

According to the results of Li et al. (2013), the average contents of Cu, Pb, Zn, Cr and As in topsoil in the Shenyang Tiexi Industrial District were 92.45, 116.76, 234.80, 67.90 and 22.69 mg/kg, respectively. The mean values of Cu, Pb, Zn and As in this study were lower than this result but the values of Cr from this study were slightly higher. Zhang (2001) studied seven heavy metals in soil in the Shenyang suburb agricultural production area. The results showed the contents of heavy metals as: As = 11.96 mg/kg, Cr = 96.2 mg/kg, Cu = 43.7 mg/kg, Pb = 102 mg/kg and Zn = 52.7 mg/kg, of which the values of As and Zn were lower than that of this study.

Shenyang industrial district has been abandoned for many years, but still there are many people live there. So it is necessary to investigate the soil heavy metal pollution characteristics and ecological risk, in order to determine whether they affect the health of local people.

The previous researchers were only focused on one aspect, such as pollutant concentration distribution or ecological risk, while this study put emphasis on various factors, which include pollution evaluation (surface soil pollution assessment, correlation of heavy metal content and clustering analysis, distribution of heavy metal content in soil profile and enrichment factor etc.) and risk assessment.

The primary objectives of this study were: (1)to determine the spatial distributions and total concentrations of titanium (Ti), copper (Cu), lead (Pb), zinc (Zn), cobalt (Co), nickel (Ni), chromium (Cr) and arsenic (As) in the Shenyang industrial district; (2)to reveal the complicated relationships between heavy metals and soil properties using correlation analysis; (3)to discriminate heavy metals as from natural or anthropogenic sources by using clustering analysis; (4)to identify anthropogenic contamination of heavy metal in topsoil using the Enrichment factor (EF); (5)to estimate the ecological risks of soil heavy metals in the study area according to Hakanson's (1980) methodology.

## Materials and Methods

### Study Site

This study was carried out in southwest Shenyang (122°25′–123°48′E, 41°11′–43°2′N). Shenyang has a total area of 13,000 km^2^ and a population of >8.23 million. It is the economic, cultural, financial and commercial center of Liaoning province, in Northeast China. It has plains, mountains and hills in the southeast and the Liaohe river, Hunhe river and Xiushui river pass through the area. Shenyang belongs to the temperate zone monsoon climate with an annual average temperature of 8.3°C, an annual rainfall of 500 mm and a frost-free period of 183 days.

### Soil Sampling and Analysis

In September and October 2011, samples were collected by a systematic sampling strategy in southwest Shenyang, using a global positioning system (GPS) to identify each location (S1 Fig). Forty-two topsoil samples were randomly distributed in the study area, based on a 700×700 m grid. Six typical soil profiles were selected to investigate the impacts of human activities on heavy metals in the soil. To avoid sampling of field paths, field ditches and other artificial buildings, sampling points were selected around designated sampling nodes, and each grid had at least one sampling point.

The main soil type was meadow brown soil, with a loam texture. Food crops were mainly corn and vegetables. The samples were air-dried at room temperature, and sieved to 2 mm to remove plant roots, large debris, and gravel-sized material. The sieved soil samples were then ground with a pestle and mortar until all particles passed through a 0.149 mm mesh.

Soil samples underwent acid digestion with a mixture of HNO_3_-HF-HClO_4_ followed by Inductively Coupled Plasma Atomic Emission Spectrometry to determine contents of Ti, Cu, Pb, Zn, Co, Ni, Cr and As. A blank reagent and standard reference material GSS-1 soil (China National Center for Standard Materials) were included for quality assurance and quality control (one blank and one standard for 10 samples).

(Ethics Statement: This study was approved by the College of Water Sciences of the Beijing Normal University. All authors provided written informed consent. For the locations/activities required no specific permissions, since the soil samples were collected from an abandoned industrial area. The soil samples are topsoil, so there is no effect on the surrounding environment, and there are no endangered or protected species around.)

## Results and Discussion

### Topsoil Pollution Assessment in the Study Area

The spatial distribution of heavy metal concentrations in topsoil in the Shenyang industrial district are separately shown in S2 Fig The trends for Cu, Pb, Zn and Cr are similar with higher concentrations between the Xihe and Hunhe Rivers, indicating that they had the same sources; most likely heavy metal emission from the Jinshan thermal power plant. Co and Ni showed very similar spatial patterns, with contamination hotspots in the middle and southwest of the area. On the south bank of the Hunhe River, the concentration of Ti was higher, possibly from sewage irrigation. High concentrations of As were in the eastern part of the study area, possibly from human sources, such as the arsenic smelting plants.

Started in the 1930s, the Shenyang smelting plant was one of the leading enterprises of China's metallurgical industry. Although now closed down, a long time is still required to completely remove the heavy metal pollution in the area. Simultaneously, the relationship between land use and soil pollution requires attention. On the whole, the land use types of the study area include paddy fields, dry land, industrial land and urban land. Farmland accounts for a significant proportion of land in the study area and has a long history of sewage irrigation which is likely the reason that heavy metal contaminated hotspots are mainly distributed in farmland.

Descriptive statistics of the eight heavy metal contents and selected topsoil properties are presented in S1 Table. The mean value of the eight heavy metal contents in the soils followed a descending order: Ti>Cr>Zn>Pb>Cu>Ni>As>Co and were all higher than their background values (Ti = 4769.31>3800 mg/kg, Cu = 33.75>22.6 mg/kg, Pb = 45.95>26 mg/kg, Zn = 81.54>74.2 mg/kg, Co = 12.91>12.7 mg/kg, Ni = 32.26>26.9 mg/kg, Cr = 83.36>61 mg/kg and As = 13.69>11.2 mg/kg) but did not exceed their pollution limits based on the Chinese Environmental Quality Standard for Soils (State Environmental Protection Administration of China, 1995). These heavy metals require further monitoring and protection measures to prevent additional enrichment[12]. The mean values of Al_2_O_3_, Fe_2_O_3_, CaO, MgO and Na_2_O in soil were 15.78, 5.61, 1.44, 1.63 and 2.03%, respectively.

The average farmland heavy metal concentration in the Shenyang area was lower than the background value[13]. The mean heavy metal concentrations from other researchers were almost all higher than the background value[14–16] but there was a large difference between studies. The large industrial zone in the Shenyang area and the different land use types led to greater differences in soil heavy metal content in different areas.

### Vertical Distribution of Heavy Metals in Typical Soil Profiles

S3 Fig illustrates the concentration distribution of eight heavy metals with depth in them. Generally, heavy metal concentrations fluctuated with increasing depth. Heavy metal concentrations decreased with soil depth in profiles SP3–SP6. Metal accumulation in deeper soils might be caused by metal mobility and soil texture[17]. Compared with 30–40 cm in SP2, heavy metal concentration was higher at 40–50 cm, which was the same as the As concentration trend in SP6. Heavy metal concentrations in SP2 were lower than the values in SP1 and SP3–SP6, indicating only slight pollution. Comparatively, accumulation peaks were observed at 20–30 cm in SP 1 for Ti, Cu, Pb, Zn, Co, Cr and As. The same trend appears in SP 5 for Cu and Pb. This might be induced by contaminants from mines and industrial sewage irrigation. Additionally, the retention of heavy metals from sewage irrigation might be the reason for higher heavy metal concentration in surface soils.

The sewage irrigation, use of pesticide and fertilizer containing heavy metals and atmospheric pollutant deposition caused the heavy metal enrichment in the soil. Heavy metals of pesticides and fertilizers remain as residues in the soil for a long time as they are easily influenced by soil organic matter and inorganic matter adsorption, leading to the formation of insoluble substances. Soil microorganisms cannot degrade heavy metal pollutants making it difficult to eliminate soil contaminants in the short-term.

### Correlation and Clustering Analysis

Correlation analysis provides an effective way to reveal the relationships among heavy metals in soil and have been helpful for understanding the influencing factors. To further investigate the interrelationships between heavy metals and soil properties, Pearson correlation coefficients were calculated and the matrix is shown in S2 Table.

On the whole, all the metal pairs showed positive relations. Ti, Co, Ni and Cr showed significantly positive correlations with total Fe_2_O_3_ (r = 0.626**-0.875**), suggesting their strong association with soil Fe oxides which are important products of parent rock weathering. Correlation coefficients of Cu-MgO (0.646**), Co-MgO (0.797**) and Cr-MgO (0.833**) suggested significant correlations with each other. As had a significant correlation (r = 0.583**) with total Al_2_O_3_, showing its strong association with aluminosilicate phases. Sand, Clay and heavy metal did not correlate with each other. The pairs of Co-OM (0.611**), Cr-OM (0.539**) and Pb-OM (0.510**) showed good relations with each other at the same confidence level. Cu exhibited a high significant positive relationship with Pb (0.755**), Zn (0.882**), Co (0.797**), Ni (0.735**) and Cr (0.878**). Cr also showed positive correlations with Ti (0.552**), Pb (0.531**), Zn (0.756**), Co (0.876**) and Ni (0.845**). Among all the correlation coefficients, the pairs of Cu-Zn, Co-Cr and Cr-Cu reached the highest at 0.882, 0.876 and 0.878, respectively. The pairs of Ni-Co, Ni-Ti, Pb-Zn reached 0.830, 0.780 and 0.791, respectively, also showing a positive correlation.

These results indicated that an original relationship between heavy metals exists, and suggested different possible heavy metal sources. Ti, Co, Ni and Cr showed significant positive correlations with total Fe_2_O_3_ (r = 0.626**-0.875**) and Cu, Pb, Cr and Co showed positive correlations with OM (r = 0.509**-0.611**), suggesting their strong association with each other.

To discriminate distinct groups of heavy metals as natural or anthropogenically sourced, an explorative hierarchical cluster analysis was performed. Geochemical association of heavy metals in the topsoil was mainly restricted to local environmental features, geological processes, and the characteristics of heavy metals[18]. S4 Fig is the cluster analysis for the eight heavy metals and two main groups of elements were obtained. The first group included Cu, Pb, Zn and As and the second group included Co, Cr, Ni, and Ti. This suggested that Cu, Pb, Zn and As may come from the same source and the local industrial activities contributed greatly to the soil contamination.

According to Goldschmidt’s geochemical classification (1954), Cu, Pb, Zn and As are in the chalcophile group and Co, Cr, Ni and Ti are in the siderophile group. This study area is an important mineralized region of base metals (Cu, Pb and Zn), so the geochemical association of trace elements was firstly controlled by geogenic process. In addition, trace element assemblage was influenced by mining and processing activities dominated by an anthropogenic input because in the process of metal mining and extracting, some sulfides would be oxidized to release As, Cu, Pb, Zn into the environment[19].

### Enrichment Factors

Enrichment factor (EF) is an important indicator which reflects the disturbance degree of human activities on the natural environment. It is feasible to apply an EF to determine the anthropogenic contamination of heavy metals in topsoil. Reference elements are often introduced for standardization to calculate the EF. Considering the crust-dominated element and choosing Al as the reference element, the factored formula can be expressed as:

EF=(CiCAl)sample(CiCAl)background (1)

Where (C_i_/C_Al_)_sample_ is the ratio of element concentration being determined (C_i_) to that of Al (C_Al_) in the topsoil sample and (C_i_/C_Al_)_background_ is the ratio in the reference Earth’s crust. Sutherland firstly proposed a five-category system [20] and according to this approach, this study proposed six classes of EF, which are given in S3 Table. The EF values and class for eight heavy metals were calculated as concentration ratios between the topsoil and background value (S4 Table.).

The EF values are <1 for Zn and Co in topsoil in the study area. The number of EF values that = 1 for Zn and Co were 9 and 8, indicating no enrichment (contamination). The Ti, Ni, and Cr levels in the topsoil samples were characterized by EF values <2, reflecting minimal contamination signals. Classes for Cu, Pb and As topsoil contamination are given priority to 1, suggesting minimal contamination. In some areas, classes for Cu, Pb and As reached 2 (moderate contamination), which shows a strong anthropogenic disturbance. Industrial and mining areas of slightly high EF values show a strong human disturbance.

From the above analysis, it is shown that there is slight heavy metal pollution in topsoil in parts of the study area. Although the EFs for Co and Zn were very small, they are an indicator of heavy metal accumulation. Some small enrichments may arise from differences in the composition of local soil material and the Earth’s crust[21,22]. Heavy metal pollution was mainly distributed in the industrial and mining areas, showing a close relationship with human activities. Some individual EF values for Pb and As were >2 but there were no sampling points of significant contamination in the study area.

It is worth noting that EFs only reveal the disturbance status of soil heavy metals and do not completely represent the pollution status and characteristics. Therefore, in the study of these elements with soil pollution characteristics, research should be combined with elements of the environmental geochemical and biogeochemical behavior, ensuring comprehensive and reliable information.

### Ecological Risk Assessment

According to Hakanson’s (1980) methodology, the potential ecological risk of a given contaminant is defined as:

Eri=Tri×Cfi=Tri×(CsiCni) (2)

$$\text{RI}=\sum_{i=1}^{n}E_{r}^{i}=\sum_{i=1}^{n}T_{r}^{i}\times \frac{C_{s}^{i}}{C_{n}^{i}}$$ (3)

Where ${\text{T}}_{\text{r}}^{\text{i}}$ is the toxic-response factor for the given element, which mainly reflects the heavy metal toxicity level and the degree of environment sensitivity to heavy metal pollution; ${\text{C}}_{\text{f}}^{\text{i}}$ is the contamination factor; ${\text{C}}_{\text{s}}^{\text{i}}$ is the measured concentration for heavy metal i; ${\text{C}}_{\text{n}}^{\text{i}}$ is the evaluation of reference value for heavy metal i. This method considers a variety of factors such as the multi-element synergy, toxicity level, pollution concentration and sensitivity to heavy metal pollution of the environment, which are widely used in environmental risk assessments.

The classification standard of ${\text{E}}_{\text{r}}^{\text{i}}$ and ecological risk index (RI) is shown in S5 Table. According to the approach of Hakanson (1980) and Xu et al. (2008), the toxic-response factor for the eight heavy metals is shown in S6 Table.

S7 Table. shows the potential RI for each topsoil heavy metal in the study area. The mean RI of the eight heavy metals in topsoil in the study area was 53.61, belonging to slight ecological risk. The potential RI of eight elements were in the order: As>Pb >Cu> Ni> Co> Cr >Ti> Zn.

Although the ecological risk of the heavy metals were generally lower, there were some areas where the heavy metal content meant a high ecological risk[23,24]. The Yuhong district is the urban land use and the high ecological risk values of Cu, Pb, Zn, Co and Cr appeared here showing that urbanization is an important cause of high ecological risk. Ni and As were mainly affected by the wastewater irrigation history of the Zhangshi and Shenfu irrigation areas and the Tiexi industrial pollution. A large amount of industrial wastewater and urban sewage was discharged into HunHe and Xihe Rivers. The resultant polluted water was used to irrigate rice in the Zhangshi and Shenfu irrigation areas and the paddy soil and crops were polluted. Although sewage in the study area has been treated in recent years, the situation has not improved significantly.

In general, the environmental quality of heavy metals in surface soil in the study area is better. The ecological risk assessment not only considered the human factors that caused the surface soil heavy metal accumulation since industrialization, but also considered the influence of the biological toxicity of different elements, thus it provides more thorough information for scientific decision-making from the perspective of crop safety and understanding of heavy metal pollution.

## Conclusions

This study identified the soil heavy metal pollution characteristics and risk assessment of the Shenyang industrial district in northeast of China and determined the spatial distributions of Ti, Cu, Pb, Zn, Co, Ni, Cr and As. There were many hotspots contaminated with Cu, Pb, Zn and Cr, suggesting human causes such as smelting plants and sewage irrigation. Soil in the study area could be considered lightly or partially polluted by heavy metals because average values were greater than the corresponding background values, but less than the guidance values for acid soil in China.

Relatively high EF values show human disturbance. According to the mean RI value of the eight heavy metals in topsoil, soils of the study area had a slight ecological risk. Based on the potential ecological risk factor, the eight elements followed this order: As>Pb >Cu> Ni> Co> Cr >Ti> Zn. In general, the environmental quality of heavy metals in surface soil in the study area is better. The explorative hierarchical cluster analysis showed that the eight elements were classified two distinct groups. Further investigation and comprehensive research of heavy metal speciation and its bioavailability is required to reduce pollution in the study area.

## Supporting Information

S1 Fig The study area and the spatial pattern of the sampling sites. (TIF)

S2 Fig The spatial distribution (TIF)

S3 Fig Vertical distribution (TIF)

S4 Fig Dendrogram obtained by cluster analysis for heavy metal contents. (TIF)

S1 Table Descriptive statistics for heavy metal contents and selected soil properties in topsoil (0–20 cm) in the study area (mg/kg for Ti, Cu, Pb, Zn, Co, Ni, Cr and As; % for Al_2_O_3_, Fe_2_O_3_, CaO, MgO, Na_2_O, OM; cmol/kg for CEC) (DOCX)

S2 Table Correlation coefficients between heavy metals and selected soil physicochemical properties. (DOCX)

S3 Table Class of enrichment factor (EF) (DOCX)

S4 Table Enrichment factor and class in topsoil in the study area (DOCX)

S5 TableClassification standard of $E_{r}^{i}$ and RI(DOCX)

S6 TableReference $C_{n}^{i}$ and toxic coefficient $T_{r}^{i}$ of different heavy metals(DOCX)

S7 Table The potential ecological risk factor for each topsoil heavy metal in the study area (DOCX)
