# Supplementary figures and images for: Soil Heavy Metal Pollution and Risk Assessment in Shenyang Industrial District, Northeast China
Source: PLoS One. 2015 May 21;10(5):e0127736. doi: 10.1371/journal.pone.0127736 (PMC4440741; doi:10.1371/journal.pone.0127736)

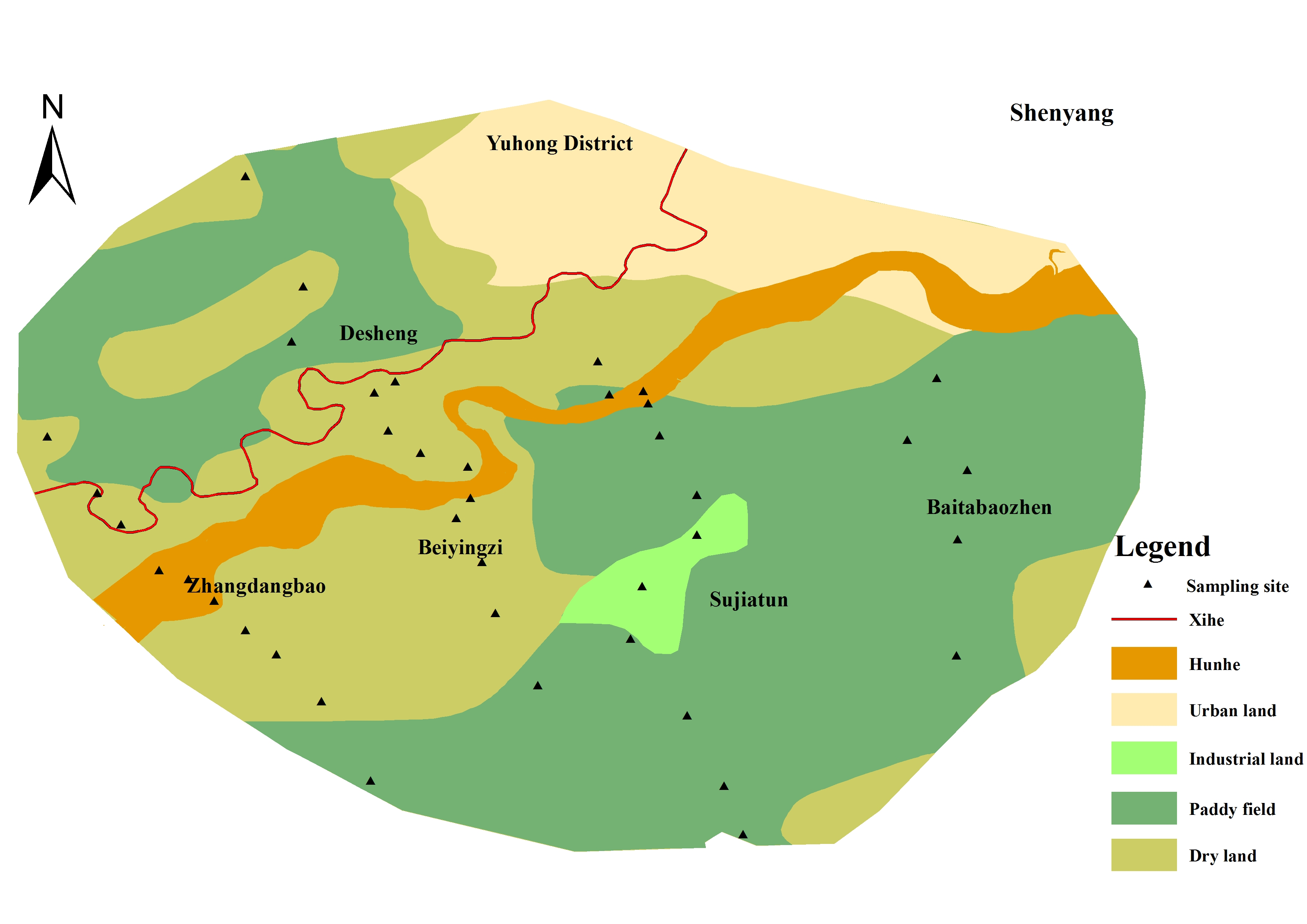

Supplement: S1 Fig — (TIF) [file pone.0127736.s001.tif]

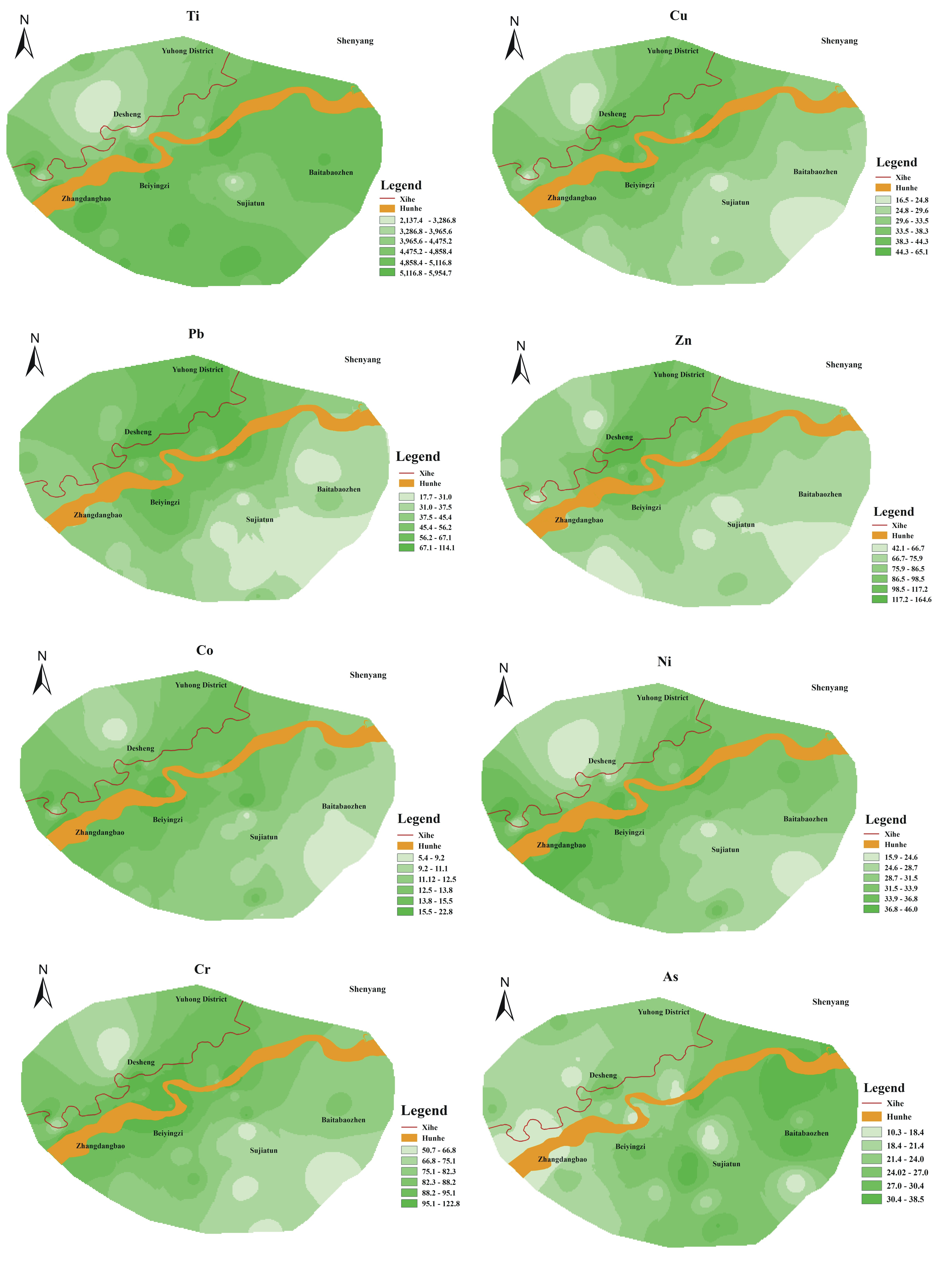

Supplement: S2 Fig — (TIF) [file pone.0127736.s002.tif]

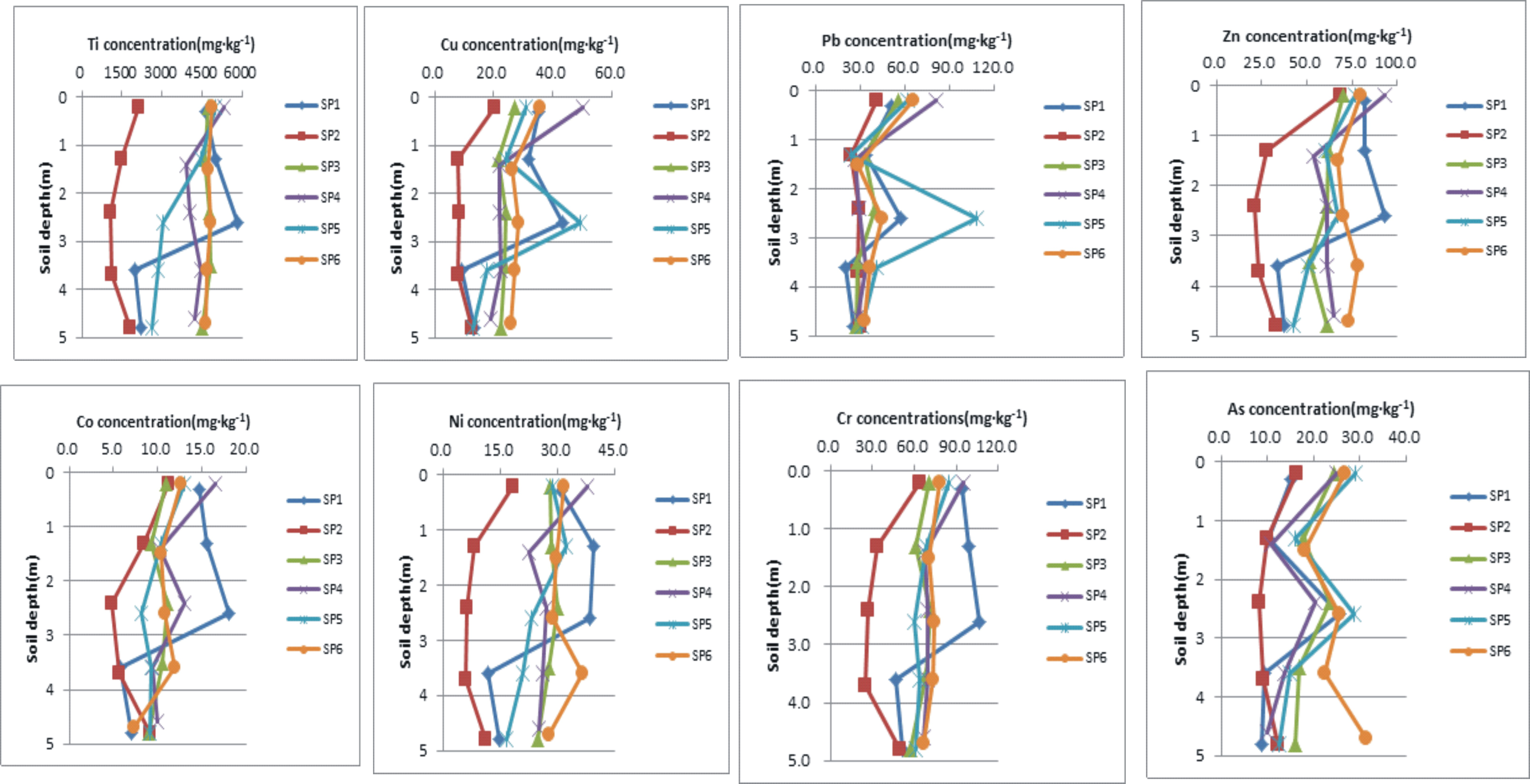

Supplement: S3 Fig — (TIF) [file pone.0127736.s003.tif]

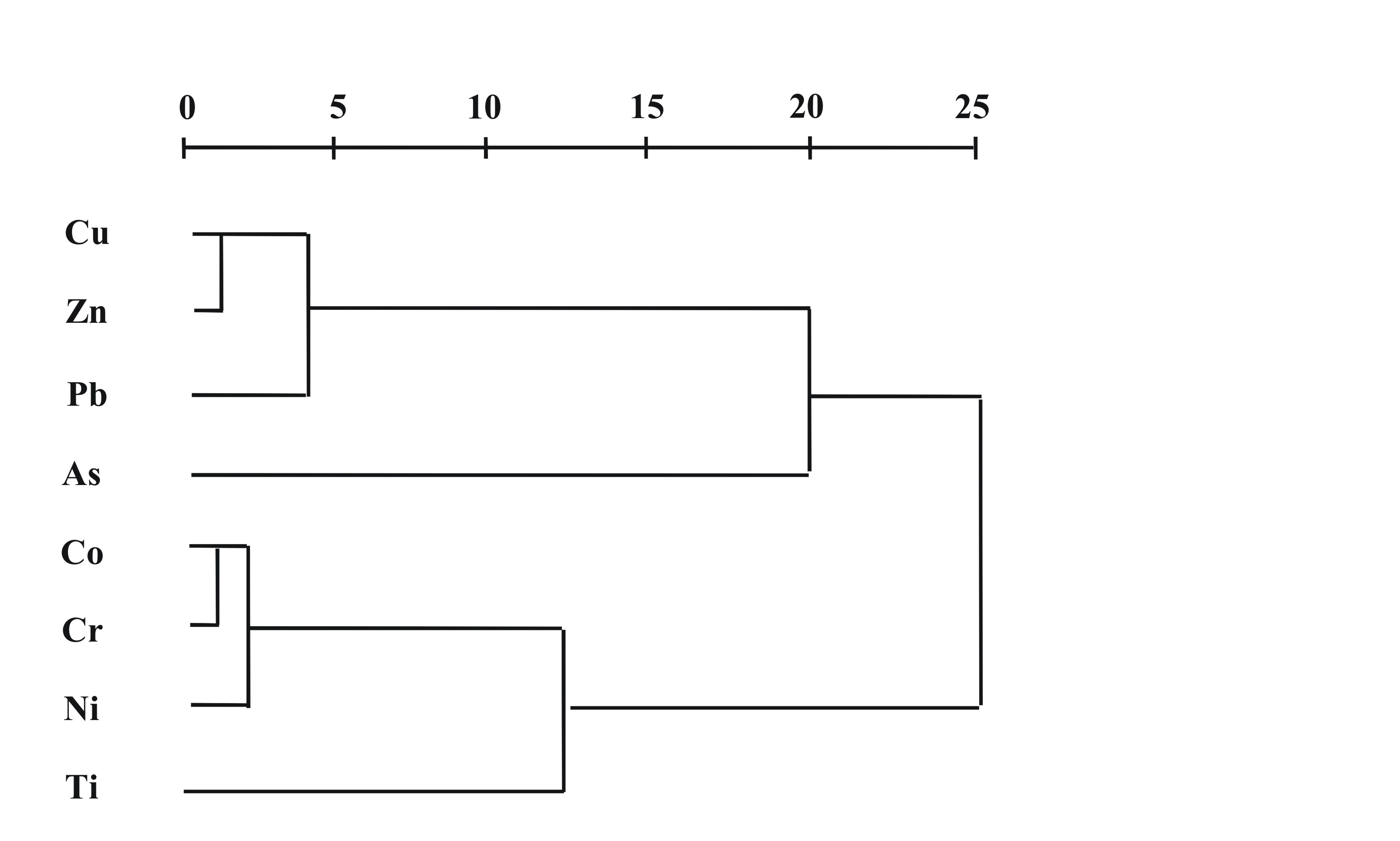

Supplement: S4 Fig — (TIF) [file pone.0127736.s004.tif]
