# Supplementary material for: Soil Heavy Metal Pollution and Risk Assessment in Shenyang Industrial District, Northeast China
Source: PLoS One. 2015 May 21;10(5):e0127736. doi: 10.1371/journal.pone.0127736 (PMC4440741; doi:10.1371/journal.pone.0127736)
Supplement: S1 Table — (DOCX) [file pone.0127736.s005.docx]

**S1 Table.** Descriptive statistics for heavy metal contents and selected soil properties in topsoil (0–20 cm) in the study area (mg/kg for Ti, Cu, Pb, Zn, Co, Ni, Cr and As; % for Al_2_O_3_, Fe_2_O_3_, CaO, MgO, Na_2_O, OM; cmol/kg for CEC)

|  | Min | Max | Mean | S D | Skewness | Kurtosis | Background value^a^ | Limit value^b^ |
| --- | --- | --- | --- | --- | --- | --- | --- | --- |
| Ti | 2136.00 | 5957.00 | 4769.31 | 826.05 | -1.72 | 3.12 | 3800 | - |
| Cu | 16.50 | 65.20 | 33.75 | 10.88 | 1.02 | 1.00 | 22.6 | 100 |
| Pb | 17.70 | 114.00 | 45.95 | 23.41 | 1.42 | 1.76 | 26 | 300 |
| Zn | 42.00 | 165.00 | 81.54 | 25.75 | 1.54 | 2.60 | 74.2 | 250 |
| Co | 5.40 | 22.80 | 12.91 | 3.39 | 0.53 | 0.95 | 12.7 | - |
| Ni | 15.90 | 46.00 | 32.26 | 7.30 | -0.15 | -0.15 | 26.9 | 50 |
| Cr | 50.60 | 123.00 | 83.36 | 16.25 | 0.25 | 0.22 | 61 | 200 |
| As | 10.20 | 38.60 | 13.69 | 7.41 | 0.10 | -0.86 | 11.2 | 30 |
| Al_2_O_3_ | 14.16 | 18.49 | 15.78 | 1.22 | 0.74 | -0.19 | - | - |
| Fe_2_O_3_ | 3.25 | 7.09 | 5.61 | 0.90 | -0.85 | 0.16 | - | - |
| CaO | 0.94 | 2.39 | 1.44 | 0.39 | 0.65 | -0.61 | - | - |
| MgO | 0.91 | 2.38 | 1.63 | 0.38 | -0.04 | -0.88 | - | - |
| Na_2_O | 1.08 | 3.37 | 2.03 | 0.62 | 0.27 | -0.89 | - | - |
| CEC | 9.80 | 31.70 | 16.54 | 5.26 | 1.33 | 1.17 | - | - |
| OM | 0.13 | 2.19 | 1.03 | 0.52 | 0.09 | -0.86 | - | - |

SD = standard deviation

a China soil background values (Wang, Wei et al.,1995)

b Chinese Environmental Quality Standard for Soils (State Environmental Protection Administration of China, 1995).
