# Supplementary material for: Soil Heavy Metal Pollution and Risk Assessment in Shenyang Industrial District, Northeast China
Source: PLoS One. 2015 May 21;10(5):e0127736. doi: 10.1371/journal.pone.0127736 (PMC4440741; doi:10.1371/journal.pone.0127736)
Supplement: S2 Table — (DOCX) [file pone.0127736.s006.docx]

**S2 Table.** Correlation coefficients between heavy metals and selected soil physicochemical properties.

|  | Ti | Cu | Pb | Zn | Co | Ni | Cr | As | Al_2_O_3_ | Fe_2_O_3_ | CaO | MgO | Na_2_O | CEC | OM | sand | clay |
| --- | --- | --- | --- | --- | --- | --- | --- | --- | --- | --- | --- | --- | --- | --- | --- | --- | --- |
| Ti | 1 |  |  |  |  |  |  |  |  |  |  |  |  |  |  |  |  |
| Cu | 0.478** | 1 |  |  |  |  |  |  |  |  |  |  |  |  |  |  |  |
| Pb | 0.062 | 0.755** | 1 |  |  |  |  |  |  |  |  |  |  |  |  |  |  |
| Zn | 0.216 | 0.882** | 0.791** | 1 |  |  |  |  |  |  |  |  |  |  |  |  |  |
| Co | 0.521** | 0.797** | 0.414** | 0.616** | 1 |  |  |  |  |  |  |  |  |  |  |  |  |
| Ni | 0.780** | 0.735** | 0.262 | 0.465** | 0.830** | 1 |  |  |  |  |  |  |  |  |  |  |  |
| Cr | 0.552** | 0.878** | 0.531** | 0.756** | 0.876** | 0.845** | 1 |  |  |  |  |  |  |  |  |  |  |
| As | 0.393* | 0.330* | 0.252 | 0.287 | 0.059 | 0.212 | 0.141 | 1 |  |  |  |  |  |  |  |  |  |
| Al_2_O_3_ | 0.456** | 0.068 | -0.251 | -0.135 | 0.070 | 0.416** | 0.173 | 0.583** | 1 |  |  |  |  |  |  |  |  |
| Fe_2_O_3_ | 0.829** | 0.525** | 0.035 | 0.281 | 0.626** | 0.875** | 0.692** | 0.374* | 0.634** | 1 |  |  |  |  |  |  |  |
| CaO | -0.346* | 0.309* | 0.431** | 0.469** | 0.284 | 0.013 | 0.339* | -0.443** | -0.604** | -0.198 | 1 |  |  |  |  |  |  |
| MgO | 0.585** | 0.646* | 0.293 | 0.495** | 0.797** | -0.804** | 0.833** | -0.149 | 0.001 | 0.691** | 0.395** | 1 |  |  |  |  |  |
| Na_2_O | -0.609** | -0.020 | 0.280 | 0.194 | -0.055 | -0.377* | -0.048 | -0.623** | -0.845** | -0.560** | 0.864** | 0.091 | 1 |  |  |  |  |
| CEC | 0.342* | 0.086 | -0.108 | -0.006 | 0.204 | 0.317 | 0.172 | 0.163 | 0.261 | 0.379* | -0.238 | 0.233 | -0.364* | 1 |  |  |  |
| OM | 0.070 | 0.509** | 0.510** | 0.456** | 0.611** | 0.431** | 0.539** | 0.072 | 0.073 | 0.179 | 0.285 | 0.299 | -0.016 | 0.155 | 1 |  |  |
| sand | -0.338 | -0.186 | 0.014 | -0.157 | -0.229 | -0.258 | -0.151 | -0.248 | -0.172 | -0.175 | 0.330 | -0.045 | 0.392* | -0.293 | -0.190 | 1 |  |
| clay | -0.056 | 0.102 | -0.035 | 0.077 | 0.181 | 0.105 | 0.178 | -0.071 | 0.094 | 0.012 | 0.032 | 0.143 | -0.002 | -0.045 | 0.201 | -0.525** | 1 |

* Significant correlation at P = 0.05.

** Extremely significant correlation at P = 0.01.
