# Supplementary material for: Soil Heavy Metal Pollution and Risk Assessment in Shenyang Industrial District, Northeast China
Source: PLoS One. 2015 May 21;10(5):e0127736. doi: 10.1371/journal.pone.0127736 (PMC4440741; doi:10.1371/journal.pone.0127736)
Supplement: S3 Table — (DOCX) [file pone.0127736.s007.docx]

**S3 Table.** Class of enrichment factor (EF)

| EF | Class | Enrichment (Contamination) level |
| --- | --- | --- |
| < 1 | 0 | no enrichment (Contamination) |
| 1-2 | 1 | deficiency to minimal enrichment (Contamination) |
| 2–5 | 2 | moderate enrichment (Contamination) |
| 5–20 | 3 | significant enrichment (Contamination) |
| 20–40 | 4 | very high enrichment (Contamination) |
| > 40 | 5 | extremely high enrichment (Contamination) |
