# Supplementary material for: Soil Heavy Metal Pollution and Risk Assessment in Shenyang Industrial District, Northeast China
Source: PLoS One. 2015 May 21;10(5):e0127736. doi: 10.1371/journal.pone.0127736 (PMC4440741; doi:10.1371/journal.pone.0127736)
Supplement: S4 Table — (DOCX) [file pone.0127736.s008.docx]

**S4 Table.** Enrichment factor and class in topsoil in the study area

|  | Ti | Cu | Pb | Zn | Co | Ni | Cr | As |
| --- | --- | --- | --- | --- | --- | --- | --- | --- |
| The number of Class 0/1 | 42 | 40 | 35 | 42 | 42 | 42 | 42 | 30 |
| The number of Class 2 |  | 2 | 7 |  |  |  |  | 12 |
| The number of Class 3 |  |  |  |  |  |  |  |  |
| The number of Class 4 |  |  |  |  |  |  |  |  |
| The number of Class 5 |  |  |  |  |  |  |  |  |
