# Supplementary material for: Soil Heavy Metal Pollution and Risk Assessment in Shenyang Industrial District, Northeast China
Source: PLoS One. 2015 May 21;10(5):e0127736. doi: 10.1371/journal.pone.0127736 (PMC4440741; doi:10.1371/journal.pone.0127736)
Supplement: S5 Table — (DOCX) [file pone.0127736.s009.docx]

**S5 Table.** Classification standard of $E_{r}^{i}$ and RI

| $E_{r}^{i}$ | Single pollutant degree of environmental risk | RI | Comprehensive environmental risk level |
| --- | --- | --- | --- |
| $E_{r}^{i}$≤40 | low ecological risk | RI ≤150 | low ecological risk |
| 40 <$E_{r}^{i}$ ≤80 | moderate ecological risk | 150 < RI ≤300 | moderate ecological risk |
| 80 <$E_{r}^{i}$ ≤160 | considerable ecological risk | 300< RI ≤600 | considerable ecological risk |
| 160 < $E_{r}^{i}$ ≤320 | high ecological risk | RI >600 | very high ecological risk |
| $E_{r}^{i}$> 320 | very high ecological risk |  |  |
