# Supplementary material for: Soil Heavy Metal Pollution and Risk Assessment in Shenyang Industrial District, Northeast China
Source: PLoS One. 2015 May 21;10(5):e0127736. doi: 10.1371/journal.pone.0127736 (PMC4440741; doi:10.1371/journal.pone.0127736)
Supplement: S6 Table — (DOCX) [file pone.0127736.s010.docx]

**S6 Table.** Reference $C_{n}^{i}$ and toxic coefficient $T_{r}^{i}$ of different heavy metals

| Element | Ti | Cu | Pb | Zn | Co | Ni | Cr | As |
| --- | --- | --- | --- | --- | --- | --- | --- | --- |
| $C_{n}^{i}$/mg·kg^-1^ | 3800 | 22.6 | 26 | 74.2 | 12.7 | 26.9 | 61 | 11.2 |
| $T_{r}^{i}$ | 1 | 5 | 5 | 1 | 5 | 5 | 2 | 10 |
