# Supplementary material for: Soil Heavy Metal Pollution and Risk Assessment in Shenyang Industrial District, Northeast China
Source: PLoS One. 2015 May 21;10(5):e0127736. doi: 10.1371/journal.pone.0127736 (PMC4440741; doi:10.1371/journal.pone.0127736)
Supplement: S7 Table — (DOCX) [file pone.0127736.s011.docx]

**S7 Table.** The potential ecological risk factor for each topsoil heavy metal in the study area

|  | Element | Number of samples | Mean | Range | Environmental risk |
| --- | --- | --- | --- | --- | --- |
| $E_{r}^{i}$ | Ti | 42 | 1.26 | 0.56-1.57 | low ecological risk |
|  | Cu | 42 | 7.47 | 3.65-14.4 |  |
|  | Pb | 42 | 8.84 | 3.41-22 |  |
|  | Zn | 42 | 1.10 | 0.57-2.22 |  |
|  | Co | 42 | 5.08 | 2.13-8.98 |  |
|  | Ni | 42 | 6.00 | 2.95-8.55 |  |
|  | Cr | 42 | 2.73 | 1.66-4.03 |  |
|  | As | 42 | 21.14 | 9.09-34.42 |  |
| RI | | | 53.61 | 31.28-88.26 | low ecological risk |
